# Embers of the Past: Early Childhood Traumas Interact with Variation in *P2RX7* Gene Implicated in Neuroinflammation on Markers of Current Suicide Risk

**DOI:** 10.3390/ijms25020865

**Published:** 2024-01-10

**Authors:** Zsuliet Kristof, Zsofia Gal, Dora Torok, Nora Eszlari, Sara Sutori, Beata Sperlagh, Ian M. Anderson, Bill Deakin, Gyorgy Bagdy, Gabriella Juhasz, Xenia Gonda

**Affiliations:** 1 Department of Psychiatry and Psychotherapy, Semmelweis University, Balassa utca 6, 1082 Budapest, Hungary; zsuliet.kristof@gmail.com; 2 Laboratory of Molecular Pharmacology, HUN-REN Institute of Experimental Medicine, Szigony utca 43, 1083 Budapest, Hungary; sperlagh@koki.hu; 3 Department of Pharmacodynamics, Faculty of Pharmacy, Semmelweis University, Nagyvarad ter 4, 1089 Budapest, Hungary; galzsofia0216@gmail.com (Z.G.); torok.dora01@gmail.com (D.T.); eszlari.nora@semmelweis.hu (N.E.); bagdy.gyorgy@semmelweis.hu (G.B.); juhasz.gabriella@semmelweis.hu (G.J.); 4 NAP3.0 Neuropsychopharmacology Research Group, Semmelweis University, Nagyvarad ter 4, 1089 Budapest, Hungary; 5 National Centre for Suicide Research and Prevention (NASP), Department of Learning, Informatics, Management and Ethics, Karolinska Institutet, Granits väg 4, 17165 Solna, Sweden; sara.stori@ki.se; 6 Division of Neuroscience and Experimental Psychology, School of Biological Sciences, Faculty of Biological, Medical and Human Sciences, The University of Manchester and Manchester Academic Health Sciences Centre, 46 Grafton Street, Manchester M13 9NT, UK; ian.anderson@manchester.ac.uk (I.M.A.); bill.deakin@manchester.ac.uk (B.D.)

**Keywords:** *P2RX7*, suicide, stress, recent life events, childhood adversities, GxE interaction, neuroinflammation

## Abstract

Both early childhood traumatic experiences and current stress increase the risk of suicidal behaviour, in which immune activation might play a role. Previous research suggests an association between mood disorders and *P2RX7* gene encoding P2X7 receptors, which stimulate neuroinflammation. We investigated the effect of *P2RX7* variation in interaction with early childhood adversities and traumas and recent stressors on lifetime suicide attempts and current suicide risk markers. Overall, 1644 participants completed questionnaires assessing childhood adversities, recent negative life events, and provided information about previous suicide attempts and current suicide risk-related markers, including thoughts of ending their life, death, and hopelessness. Subjects were genotyped for 681 SNPs in the *P2RX7* gene, 335 of which passed quality control and were entered into logistic and linear regression models, followed by a clumping procedure to identify clumps of SNPs with a significant main and interaction effect. We identified two significant clumps with a main effect on current suicidal ideation with top SNPs *rs641940* and *rs1653613*. In interaction with childhood trauma, we identified a clump with top SNP *psy_rs11615992* and another clump on hopelessness containing *rs78473339* as index SNP. Our results suggest that *P2RX7* variation may mediate the effect of early childhood adversities and traumas on later emergence of suicide risk.

## 1. Introduction

Suicide is one of the most burning psychiatric and public health concerns, claiming more than 700,000 lives every year, and for each completed suicide, 10–20 times more attempts happen [1]. Suicide occurs throughout the lifespan, and it is among the leading causes of death in adolescents and young adults [1,2], and, alarmingly, it also shows a sharply increasing tendency in these age groups and children [3]. Underpinned by an interplay between genetic, psychological, and environmental factors [4], suicidal behaviour occurs along a spectrum from suicidal ideation due to the combination of pain and hopelessness, which escalates into planning if pain exceeds connectedness, while dispositional, learned, and practical contributors to suicide capacity facilitate the transition from ideation and planning to attempts at the other end of the spectrum [5]. Although suicidal thoughts and behaviours can be reduced by psychological treatments and pharmacotherapy (lithium, ketamine, esketamine, and clozapine) [6,7], there is no pharmacological intervention that has a sufficient established effectiveness in preventing suicide, creating an immense unmet need [2,8]. However, some studies suggest that drugs that have antisuicidal effects seem to exert anti-inflammatory action as well, and certain medications acting on the immune system may possess antisuicidal properties [4,9].

Most recent genome-wide association studies (GWAS) have suggested that polygenic risk and specific loci, novel for suicide but previously linked to psychiatric disorders and risk factors for depressive disorder, are associated with a higher risk of attempting suicide [10,11]. Long-term and recent stressful life events, such as employment and financial difficulties, loss of a loved one, and diagnosis with terminal or chronic illness can increase the incidence of suicidal behaviour [12,13]. Emerging evidence suggests that childhood maltreatment and trauma, such as sexual, physical, and emotional abuse, neglect, or exposure to domestic violence have particularly noxious effects and increase the rate of impulsive and suicidal behaviour by two to seven times [14,15,16]. We increasingly also understand that different types of stressors impact the immune system in various ways, also increasing chronic low-grade inflammation [17]. Stress is also involved in neuroinflammation as, upon stress exposure, the resident immune cells of the CNS, microglia, become activated, which leads to the release of neuroactive molecules such as adenosine triphosphate (ATP), glutamate, nitric oxide (NO), brain-derived neurotrophic factor (BDNF), and pro-inflammatory cytokines, including TNF-α and IL-1 β [18,19]. 

Disturbances in the immune system, especially inflammation, play a critical role in a range of psychiatric disorders, including schizophrenia, major depressive disorder, or bipolar disorder [20]. Several studies have suggested that inflammatory mediators are involved in the pathophysiology of suicide as well [9,21,22,23,24,25,26]. Elevated markers of inflammation, such as C-reactive protein, proinflammatory cytokine IL-1β and IL-6, and TNF-α have been reported, both in suicidal patients’ central nervous system and peripheral tissues, regardless of their primary diagnosis, age, and gender [4,27]. Moreover, studies utilizing various approaches provide evidence for the role of microglial cells in suicide [28], including the report of augmentation of microglial density or priming and macrophage recruitment in the brains of suicide victims [28,29,30]. 

Purinergic receptor P2X7 (P2X7R) is an ATP-sensitive ligand-gated nonselective cation channel, expressed in the brain primarily by microglia [31]. Activation by a marked increase in extracellular ATP leads to the NLRP3 inflammasome complex-mediated release of proinflammatory cytokines and neurotransmitters into the extracellular space [32], including IL-1 β, IL-18 and glutamate [33]. A growing body of data indicates that pharmacological or genetic blockade of P2X7 receptors could potentially elicit antidepressant- and/or anxiolytic-like effects [34,35]. The potential role of *P2RX7* in mood disorders is also underpinned by our previous studies, where we found that variation in this gene influences the severity of current depressive and anxiety symptoms in interaction with life stress, mediating the effects of both early, distal stressors and recent, proximal stressors [36,37]. 

**Figure 1:**
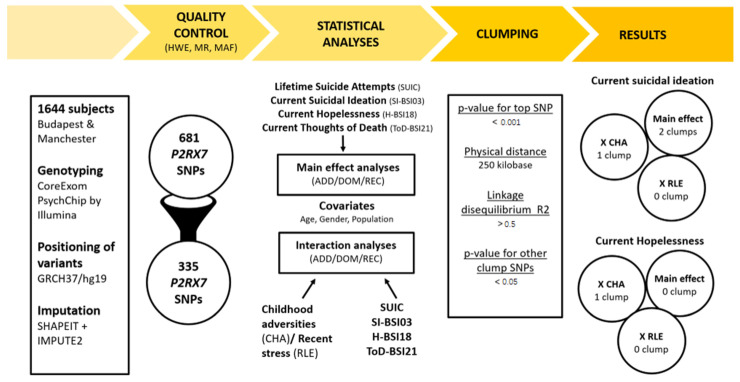
Overview of the study focusing on variation along the *P2RX7* gene in interaction with early childhood adversities and traumas and recent life events on previous suicide attempts and markers on current suicide risk.

Although several studies, including GWAS-s [38], suggest a relationship between chromosome 12q2431, where *P2RX7* gene is located, and the development of mood disorders [39,40], whose association with suicide is evident, and despite the increasing attention focusing on the role of neuroinflammation and suicide, there have been no studies investigating the involvement of the P2X7 receptor and the *P2RX7* gene in suicidal behaviour. As GWAS-s necessarily, employ overly strict *p*-value criteria in order to correct for the very high number of performed statistical tests may overlook existing associations [41,42], we decided to reduce multiple testing burden and still overcome the drawbacks of candidate variant studies by employing a clumping methodwhich focuses on all SNPs along a given gene, but “clumps” together those variants which are inherited together based on linkage disequilibrium. In our current study, we focused on the effects of variation along the *P2RX7* gene in interaction with early childhood adversities and recent negative life events on previous lifetime suicide attempts and current markers of suicidal ideation in a large general white European sample (Figure 1).

## 2. Results

### 2.1. Main Effects of Variation in P2RX7 on Lifetime Suicide Attempts, Current Suicidal Ideation, Current Hopelessness, and Current Thoughts of Death

**Figure 2:**
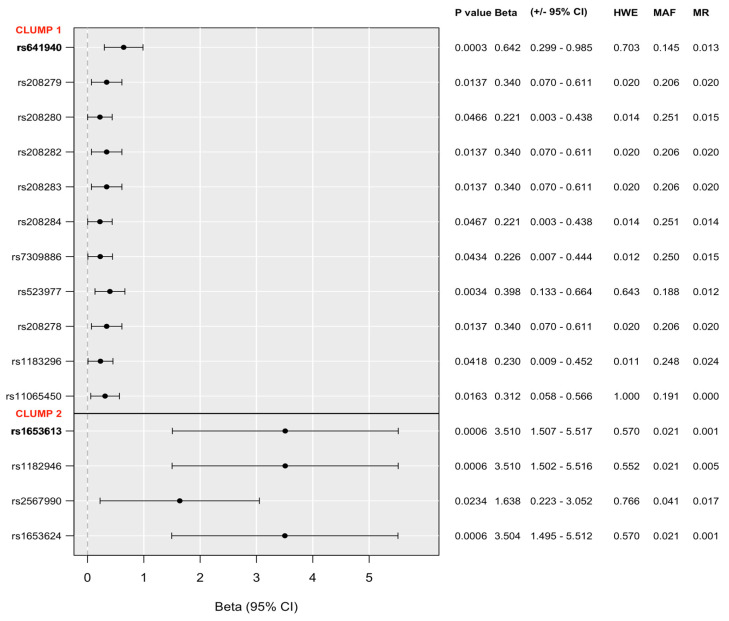
Significant clumps of *P2RX7* SNPs in models for main effect on current suicidal ideation (SI-BSI03) in the recessive model. *Bold type denotes top SNPs in each clump. 95%CI: 95% confidence interval. HWE: Hardy–Weinberg equilibrium. MAF: minor allele frequency. Missing: missingness rate*.

Logistic and linear regression models on SUIC (for previous lifetime suicide attempts), H-BSI18 (for hopelessness), and ToD-BSI21 (for thoughts of death) identified no SNPs reaching the nominal significance threshold. However, a linear regression model on SI-BSI03 (for current suicidal ideation) revealed some SNPs with a nominally significant main effect, the *p*-values of which did not exceed the maximum threshold specified for clumping (*p* = 0.001) either, thus, significant clumps could be formed. In the recessive model, one identified clump contained 12 SNPs, with *rs641940* as the top SNP and the minor C allele as a risk allele with an allele frequency of 0.1450, while another clump contained 4 SNPs, with *rs1653613* as the top SNP and the minor G allele as risk allele, the frequency of which was 0.0214 (Figure 2). No significant clumps emerged in the additive and dominant models for current suicidal ideation. Linear regression results on current suicidal ideation according to the recessive model, including all *P2RX7* SNPs in the NewMood database together with quality controls results, are shown in Supplementary Table S1. 

### 2.2. Gene x Environment Effects of Variation in P2RX7 on Lifetime Suicide Attempts, Current Suicidal Ideation, Current Hopelessness, and Current Thoughts of Death: Interaction with Childhood Adversities (CHA)

Our analyses on the interaction between *P2RX7* and childhood adversities on lifetime suicide attempts (SUIC), and on current thoughts of death (ToD-BSI21), yielded no significant clumps.

**Figure 3:**
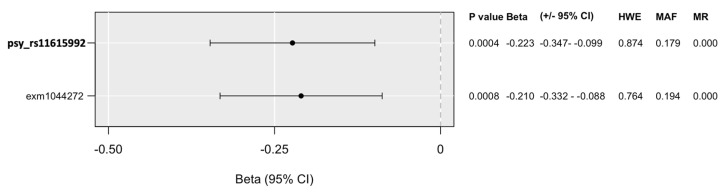
Significant clump of *P2RX7* SNPs in interaction with early childhood adversities and traumas (CHA) on current suicidal ideation (SI-BSI03) in the dominant model. *CHA: childhood adversities. Bold type denotes top SNPs in each clump. 95%CI: 95% confidence interval. HWE: Hardy–Weinberg equilibrium. MAF: minor allele frequency. Missing: missingness rate*.

Linear regression models on the interaction between *P2RX7* and childhood adversities on current suicidal ideation (SI-BSI03) yielded one significant clump, surviving correction for multiple testing. In the dominant model, the identified clump contained two SNPs, with *psy_rs11615992* as the most significant SNP (Figure 3), and the minor G allele as a protective allele, with an allele frequency of 0.1794. Linear regression results in interaction with early childhood adversities and traumas (CHA) on current suicidal ideation (SI-BSI03) according to the recessive model including all *P2RX7* SNPs in the NewMood database together with quality control results are shown in Supplementary Table S2.

**Figure 4:**
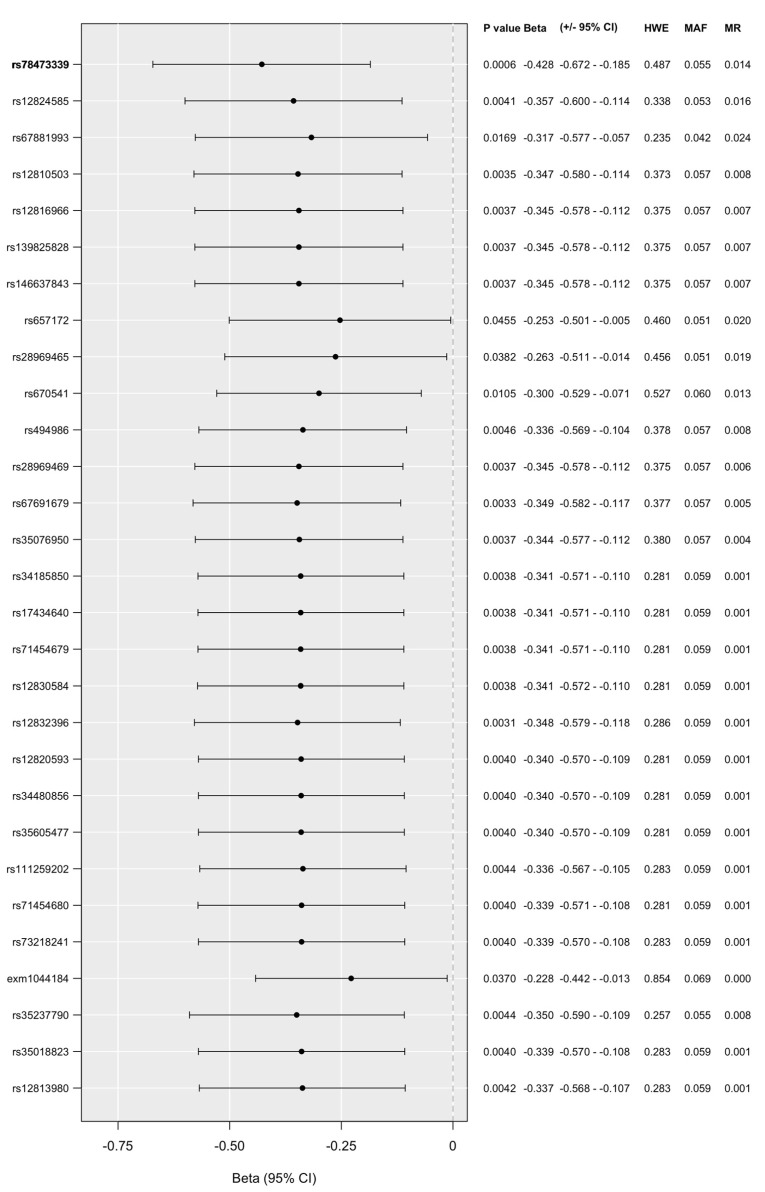
Significant clump of *P2RX7* SNPs in interaction with early childhood adversities and traumas (CHA) on hopelessness (H-BSI18) in the additive model. *CHA: childhood adversities. Bold type denotes top SNPs in each clump. 95%CI: 95% confidence interval. HWE: Hardy–Weinberg equilibrium. MAF: minor allele frequency. Missing: missingness rate*.

Linear regression models on the interaction between *P2RX7* and childhood adversities on current hopelessness (H-BSI18) yielded one clump in the additive model (Figure 4), consisting of 29 SNPs with top SNP *rs78473339* and the minor C allele as a protective allele, with an allele frequency of 0.0546. Linear regression results in interaction with early childhood adversities and traumas (CHA) on hopelessness (H-BSI18) according to the recessive model including all *P2RX7* SNPs in the NewMood database together with quality control results are shown in Supplementary Table S3.

### 2.3. Gene x Environment Effects of Variation in P2RX7 on Lifetime Suicide Attempts, Current Suicidal Ideation, Current Hopelessness, and Current Thoughts of Death: Interaction with Recent Life Events (RLE)

In case of gene x environment interaction models with recent life events on Lifetime Suicide Attempts, Current Suicidal Ideation, Current Hopelessness, and Current Thoughts of Death, logistic and linear regression models did not yield any significant clumps.

### 2.4. In Silico Characterization and Functional Prediction of Identified Top SNPs

**Figure 5:**
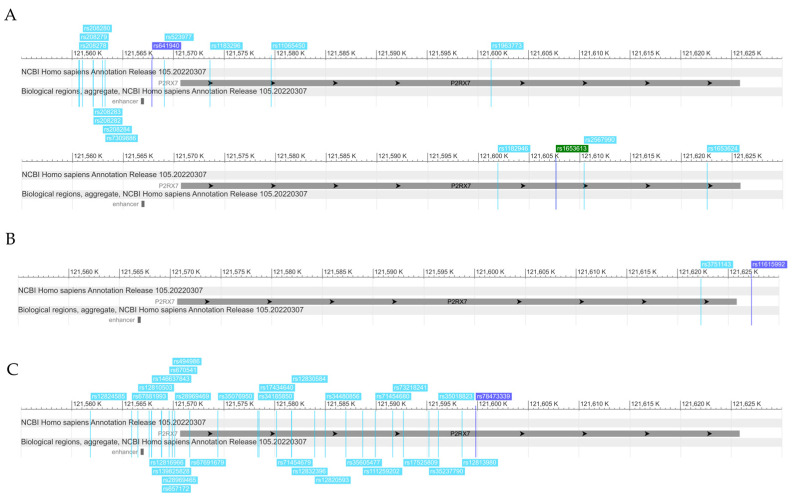
Genomic location of significant SNPs identified in the clumping procedure. (**A**) Significant clumps of *P2RX7* SNPs in models for main effect on current suicidal ideation (SI-BSI03) in the recessive model; (**B**) significant clump of *P2RX7* SNPs in interaction with early childhood adversities and traumas (CHA) on current suicidal ideation (SI-BSI03) in the dominant model; (**C**) significant clump of *P2RX7* SNPs in interaction with early childhood adversities and traumas (CHA) on hopelessness (H-BSI18) in the additive model. *Darker background colour indicates lead SNPs in all panels*.

Genomic locations of significant SNPs and top SNPs identified in the clumping procedure are shown in Figure 5.

For functional characterization of the identified top SNPs, we performed searches in LitVar, dbSNP, UCSC Genome Browser and GWAS catalog database. Of the top SNPs of each clump, *rs641940* is an intergenic variant, whereas *rs1653613* and *rs78473339* are intron variants with no known functions. *Rs11615992* is a regulatory region variant which has previously been associated with schizophrenia [43].

We also performed pathway analysis of the identified index variants with SNPNexus. SNPNexus uses Reactome data to link genes involved in the observed variants. For each pathway, a *p*-value is provided, taking into account all the genes associated with the original SNP set. The identified pathways for our top significant variants (*rs11615992*, *rs641940*, *rs78473339*, and *rs1653613*) included (1) NLRP3 inflammasome, R-HSA-844456 (*p* = 0.0013); (2) elevation of cytosolic Ca^2+^ levels, R-HSA-139853 (*p* = 0.0014); (3) inflammasomes, R-HSA-622312 (*p* = 0.0018); (4) purinergic signaling in leishmaniasis infection, R-HSA-9660826 (*p* = 0.0022); (5) cell recruitment (pro-inflammatory response), R-HSA-9664424 (*p* = 0.0022); (6) platelet calcium homeostasis, R-HSA-418360 (*p* = 0.0026); (7) nucleotide-binding domain, leucine-rich repeat-containing receptor (NLR) signaling pathway, R-HSA-168643 (*p* = 0.0050); and (8) platelet homeostasis, R-HSA-418346 (*p* = 0.0079).

## 3. Discussion

Our present study investigated the effects of variation in the *P2RX7* gene on lifetime suicide attempts and predictors of current suicidal risk, including hopelessness [44], thoughts of death [45], and suicidal ideation [46] in a large European general population sample, using a clumping procedure to investigate all available variants along the gene. We identified two clumps of variants with a main effect on current suicidal ideation (with *rs641940* and *rs1653613* as top SNPs in the recessive model), while no significant effect on lifetime suicide attempts, current hopelessness, or thoughts of death appeared. In interaction with early childhood adversities and traumas, in the case of current suicidal ideation, we identified one significant clump in a dominant model, comprising 2 SNPs with top SNP *psy_rs11615992*. In the case of hopelessness, a well-established independent predictor of suicide [44], we also identified one clump of variants interacting with childhood maltreatment in an additive model, involving 29 SNPs with *rs78473339* as the index SNP. We found no significant interaction with recent negative life events for the *P2RX7* gene on lifetime suicide attempts, or any current predictors of suicide risk. Our findings, which are the first to suggest the involvement of *P2RX7* in suicidal risk factors, not only support previous results on the potential nexus of neuroinflammation and suicidality, but also emphasise the role of a gene which has not yet been investigated in this context. Furthermore, our results add to our increasing understanding of mediators of the relationship between early childhood adversities and traumas and later emerging suicide risk. 

### 3.1. The Potential Role of Neuroinflammation, P2X7 Receptors and the P2RX7 Gene on Suicidal Behaviour

Recently, the role of neuroimmune systems has gained increased attention in psychiatric disorders and symptoms also including mood disorders and suicidality, and one of the most prominent emerging neuroinflammatory targets is the ATP-sensitive P2X7 purinergic receptor (P2X7R) due to its significant role in facilitating neuroinflammation in the central and peripheral nervous system [20,47]. The P2X7R is a ligand-gated nonselective cation channel [33] activated by elevated concentrations of extracellular ATP, which induce Na+ and Ca++ influx and K+ efflux and, as a result of repeated or sustained stimulation, a pore allowing organic ion passage including choline or N-methyl-D-glucamine (NMDG+) opens [48]. P2X7 receptors are highly expressed in various immune cells (in particular macrophages and microglia), epithelial cells, oligodendrocytes of the CNS, and Schwann cells of the PNS [49,50], while functional P2X7 expression in neurons and astrocytes is debated [51].

As a result of the wide distribution of the P2X7 receptor, its activation has a direct or indirect effect on a number of physiological and pathophysiological processes. In brief, these processes include the tonic inhibition of BDNF production in the CNS [52], regulation of cell proliferation and cell death, as well as the release of microparticles and exosomes, the production of reactive oxygen and nitrogen species and multinucleated cells, stimulation of glutamate response, the activation of the inflammasome, and the subsequent release of interleukin (IL)-1β and IL-18. [53]. Although the role of P2X7 receptors has been investigated in several neurodegenerative and psychiatric disorders, such as Parkinson’s or Alzheimer’s disease, multiple sclerosis, depression, bipolar disorder, schizophrenia, and anxiety, there has not been a study so far that explored its role in suicidal behavior.

Nevertheless, the contribution of inflammatory processes to the pathophysiology of suicide is increasingly researched [2,4,9,24,26]. Inflammatory activation of the immune system in patients with a higher suicide risk has been investigated regarding cytokines in serum, cerebrospinal fluid, and postmortem studies [54,55,56]. Suicidal patients display increased levels of proinflammatory cytokines IL-6, IL-1β, and TNF-α, [54,57], in addition to decreased neuroprotective IL-8 [26], and even a history of hospitalization due to infection has been associated with suicidal behaviour [58]. High concentrations of the acute-phase marker of inflammation, C-reactive protein (CRP), in plasma was also correlated with suicidal intent [59,60]. Systemic inflammation has been suggested to increase the risk of suicide completion as well [61]. In postmortem studies, significantly elevated mRNA and protein levels of IL-1β, IL-6, and TNF-α were observed in the prefrontal cortex of adults who committed suicide [26], while the latter marker was found to be elevated in the dorsolateral prefrontal cortex as well, regardless of psychiatric diagnosis [62,63]. In this area, decreased chemokine (CCL1, CCL13, CCL17) and anti-inflammatory IL-10 concentrations were reported in suicide completers compared to controls [63,64]. The significance of inflammation in suicide and depression is also highlighted by observations of different treatments’ outcomes. A considerable amount of patients undergoing therapy with cytokines, for example interferon-α, have been found to develop depression [64,65], suicidal ideation or attempt suicide [66,67]. Meanwhile, favorable results have been reported regarding nonsteroidal anti-inflammatory drugs (NSAID): patient populations using ibuprofen, naproxen, celecoxib, or aspirin experienced significantly less suicidal ideation compared to the ones receiving acetaminophen treatment [68,69]. 

### 3.2. P2X7 Receptors and the P2RX7 Gene as a Potential Focus of Attention in Understanding and Treatment of Neuropsychiatric Disorders and Suicide

In humans the *P2RX7* gene encoding the P2X7 receptor is located at 12q24.31 chromosome position [69], a region already implicated in affective and anxiety disorders [70]. The *P2RX7* gene comprises 13 exons and encodes an 595-amino acid protein subunit. The expressed receptor has 10 splice variants, 3 of which have been identified in humans, showing highly divergent downstream signalling properties [71,72]. Genetic variation in *P2RX7* has been shown to contribute towards gain or loss of function, leading to further variation in P2X7 receptor function [20]. Polymorphisms are widespread in the human *P2RX7* gene with at least a dozen non-synonymous polymorphisms (NS-SNPs), with consequent changes in amino acid sequence and receptor function yielding possibly altered susceptibility to various neuropsychiatric disorders [73]. Preclinical studies using genetic and pharmacological interventions implicate the P2X7 receptor as crucial in stress-induced anxiety- and depression-like behaviours in rodent models. In addition, several human studies reported significant association between *P2RX7* SNPs and NS-SNPs and vulnerability to affective disorders [74]. Although *P2RX7* has not yet emerged as a candidate gene for suicidal behaviour, in a previous GWAS many of the strongest candidate genes were associated with inflammatory response (*ADAMTS14*, *PSME2*) [75].

### 3.3. Effects of P2RX7 Variation on Suicidal Behaviour in Interaction with Childhood Adversity

Experiencing childhood abuse and/or neglect has severe negative psychological consequences that often persist into adulthood, appearing not only as high-risk behaviours, including substance abuse [76] and risky sexual behaviour [77], but in the form of substantial mental health problems, such as depression, anxiety, posttraumatic stress disorder [78,79], and suicide [80,81]. Childhood trauma has established immunological effects that may contribute to suicide risk. Major negative experiences that occur within sensitive developmental windows adversely influence the immune response, inducing elevated inflammatory states that lead to long-term consequences on both the brain and behaviour [82]. Altered dynamics of the HPA-axis, abnormal cortisol stress reactivity [83], persistently increased levels of inflammatory cytokines including IL-6 and TNFα, low-grade elevations in the inflammatory marker CRP, and greater inflammatory responses to later psychosocial stress [84] have been linked to high levels of early-life trauma. 

Psychosocial stress, which is a major environmental etiological contributor to suicide risk, has been found to be associated with changes in ATP-mediated P2X7 receptor signalling and also neuroinflammation [20]. P2X7R is expressed in various tissues and cells, including monocytes/macrophages, lymphocytes, dendritic cells [85], and microglia, the resident immune cells of the CNS [86,87], raising P2X7R to be a key driver of neuroinflammation, not only in the periphery but also in the brain [88]. In healthy tissues, under non-pathological conditions, when the extracellular concentration of ATP is low, P2X7R remains silent [32]; however, stressful circumstances evoke a marked increase in extracellular ATP, working as a damage-associated molecular pattern (DAMPs), and resulting in the activation of P2X7 receptors in microglia [89]. This leads to the activation of the NRLP3-associated inflammasome, which in turn eventuates the enhanced release of proinflammatory cytokines IL-1β and IL-18, while P2X7R activation also facilitate the release of other cytokines, such as IL-6 and TNF-α [90]. The above process has been suggested to play a significant role in the impact of chronic stress, leading to impaired neuroplasticity and the emergence of depressive-like behaviour, while pharmacological or genetic blockade of the *P2RX7*-NLRP3-IL1β pathway might foster resilience against stress via the involvement of microglia and monocytes, suggesting the potential role of P2X7R signalling as a connecting point between chronic stress and mood disorder [91,92]. 

In the present study, we revealed using a clumping method that variation along the *P2RX7* gene shows significant, both main and interaction effects with childhood adversity on current suicidal ideation, and additionally a significant interaction effect with childhood adversity on current hopelessness. As noted before, there have been no studies so far to investigate and provide results on the potential role of the *P2RX7* gene in suicidality; thus, we can examine our results in light of the previous work on neuroinflammation and suicide. A recent study investigating depressed patients found that childhood adversities and high suicide risk are associated with the upregulation of various pro-inflammatory cytokine and compound genes, including IL-1β, IL-6 and TNF, and of genes related to the adhesion, coagulation, and chemotactic ability of monocytes, while in patients lacking childhood adversities and having low suicide risk, reduced gene expression was detected [57]. As P2X7R has a significant role in processes related to the impairment of neuroplasticity as well [20], it is remarkable that early-life stress was found to regulate genes and pathways involved in neuronal plasticity differentially [93]. Genome-wide methylation studies revealed that, in individuals who committed suicide and experienced childhood disadvantages, early-life stress was linked to altered methylation of genes involved in neuronal growth and neuroprotection in the hippocampus [94,95]. Studies on peripheral tissues of survivors also underlined the association between childhood traumas and differential methylation of genes involved in neuronal plasticity [96]. Furthermore, both animal and human studies of plasticity genes, such as *BDNF* and *TRKB*, are consistent with the above findings [97,98,99]. In our previous study, we identified several clumps in the *IL6* gene influencing lifetime suicide attempts, current thoughts of death, and current suicidal ideation, but only in interaction with early childhood adversities [25], the last one of which matched the pattern seen for *P2RX7*. These data support the involvement of the immune system as a mediator between childhood adversity and increased suicide risk through a long-lasting activation of inflammation, which is complemented by our results on *P2RX7*.

Our results are also in line with animal models examining the consequences of perinatal stress on development and long-term behaviour, and the possible later effects of neuroinflammation. Perinatal stress may lead to the increased density and activity of microglial cells in the hippocampus and frontal cortex, and depression-like disruptions [100,101]. The first factor is linked to alterations in the expression of various developmental genes involved in inflammation among other processes [102,103]. As a result of maternal immune activation, the postnatal amygdala is affected by microglial cell activation as well [104]. A wide range of studies has shown that microglial function disruptions in critical developmental periods might result in structural alterations and behavioural perturbations, such as anxiety, depression, social bonding, that last into adulthood [102,105], and thus might lead to suicidal behaviour [106]. By revealing a significant main effect and interaction of childhood adversities and several variants in the *P2RX7* gene, our results highlight the role of both inflammation and altered P2X7 signalling in predisposing to suicide risk.

### 3.4. Limitations

Our study has several limitations that must be taken into account when interpreting the results. First, childhood adversities, recent life events, and lifetime suicide attempts were retrospectively evaluated based on the subjects’ self-report, which might contribute to recall and reporting bias. Similarly, current suicidal ideations, thoughts of death and hopelessness were also self-reported. Second, measuring childhood adversities and recent negative life events did not take into account the diverse severity and subjective effect of individual life events. Third, approximately two-third of our sample is female subjects and its overall scope is limited to European white participants, which may limit the generalizability of our results to the society at large. Fourth, we could not separate depression from our suicidal phenotypes, and so its contribution to suicidal behavior cannot be ruled out. However, our study is not lacking in strengths in any way: instead of hypothesis-based candidate SNP selection, we considered several hundred variants along the *P2RX7* gene with a clumping method, utilizing a dimensional approach to capture different emergences of suicidal behavior along the suicide spectrum, and using a GxE paradigm with two etiologically different types of stressors.

## 4. Methods

### 4.1. Study Population

1644 non-related volunteers (471 males, 1173 females) with self-reported European white ethnic origin aged 18–60 (mean age: 32.44 years) from Greater Manchester and Budapest were recruited between 2005 and 2008 through general practices and via an online platform to participate in the NewMood study (New Molecules in Mood Disorders, Sixth Framework Program of the European Union LHSM-CT-2004-503474). After providing written informed consent, participants provided genetic data using a saliva-based sampling kit for genotyping, and self-reported sociodemographic data including age, gender, recent negative life events occurring in the past year, childhood adversities, and suicide- and suicide risk-related markers for phenotyping. Detailed background information regarding sociodemographic background, information related to mental and somatic health and treatment received, as well as information on psychological and personality factors not used in the present analyses was collected. A more detailed description of the sample population can be found in our previously published reports [107,108,109]. The study has been conducted in accordance with the Declaration of Helsinki, and it has been approved by the Scientific and Research Ethics Committee of the Medical Research Council, Budapest, Hungary, and by the North Manchester Local Research Ethics Committee, Manchester, United Kingdom. All participants provided written informed consent prior to participation.

### 4.2. Phenotypes

Our present study focused on measuring four suicide- and suicide risk-related phenotypes, and two types of environmental stressors. 

Previous lifetime suicide attempts (SUIC) were recorded based on self-reports, producing a dichotomous variable. Current markers of suicidal risk were assessed by relevant items of the Brief Symptom Inventory [110], a questionnaire measuring psychopathological symptoms in several scales during the previous week, using a scale from 0 to 4 (“Not at all” to “Extremely”) depending on the distress caused. In case of this study, 3 items were utilized: “Thoughts of ending your life” (SI-BSI03) was used to evaluate the level of current suicidal ideation; “Feeling hopeless about the future” (H-BSI18) was used to reflect the actual level of hopelessness, a well-established independent risk factor of suicide; and “Thoughts about death and dying” (ToD-BSI21) was used to measure death-related thoughts. 

We assessed childhood adversities and traumas (CHA) as distal and etiological stressors, and recent negative life events (RLE) occurring in the past year as proximal trigger stressors, to evaluate gene–environment interactions contributing to the emergence of the measured suicidal behaviours. Early childhood adversities and traumas (CHA) were assessed by a short form of the Childhood Trauma Questionnaire (CTQ) [111], which includes four items on parental abuse and neglect, and two additional items on the loss of the parent, as validated previously [109]. In the analyses, the sum of item scores was used. Recent negative life events (RLE) occurring within the last 12 months were registered via the List of Threatening Experiences [112,113], incorporating four types of stressful life events regarding financial difficulties, illnesses/injuries, personal problems, and intimate relationship or social network difficulties. In the statistical analyses, the number of recent negative life events was used. 

### 4.3. Genotyping

In order to detect DNA, buccal mucosa cells were provided by participants using a cytology brush (Cytobrush plus C0012, Durbin PLC, Hayes, UK). Extraction of the genomic DNA was carried out according to the previously described protocol of Freeman et al. [114]. *P2RX7* genotype characterization was performed using Illumina’s CoreExom PsychChip. All laboratory work was performed under the ISO 9001:2000 quality management requirements and was blinded regarding phenotype. 

### 4.4. Statistical Analyses

In the analyses below, Plink v1.90 was used to calculate MR (<0.05), HWE (>1 × 10^−5^) and MAF (>0.01) as part of quality control steps prior to the analyses, for clumping, and for building logistic and linear regression models to test for main and interaction effects of genetic variation in the *P2RX7* gene. Analyses were supported by scripts individually written in R 3.0.2 (R Core Team, 2013). Descriptive statistics were calculated using IBM SPSS Statistics 25.

Genotyping provided a dataset comprising 1644 individuals genotyped for 681 SNPs in the region of *P2RX7* gene (with boundaries extended by 10 kb) available in the NewMood database. The 3-step quality control protocol applied for the SNPs involved the calculation of Hardy–Weinberg Equilibrium (HWE; >1 × 10^−5^), missingness rates (MF; <0.05), and minor allele frequencies (MAF; >0.01). The 335 SNPs surviving quality control steps were entered into analyses with logistic and linear regression models to investigate the main effects of *P2RX7* variation on lifetime suicidal behaviour, current suicidal ideation, current hopelessness, and current thoughts of death, which was followed by gene–environment interaction models with early childhood adversities and recent negative life events (Figure 1). 

After running the regression models, a clumping procedure followed, both for main effect and for the two types of GxE interaction effects separately, based on linkage disequilibrium (LD) estimates between the SNPs using the CLUMP function in Plink. Clumping is a statistical method for yielding clumps of intercorrelated SNPs based on empirical estimates of their linkage disequilibrium (LD), stepping beyond independent significance levels, identifying connected SNPs and their top SNP, the one with the highest significance. Four parameters were used for clumping: (1) maximum *p*-value of the clump’s top SNP was set at 0.001; (2) maximum *p*-value for the clump’s secondary SNPs was 0.05; (3) minimum LD R2 with top SNP was 0.5; and (4) physical distance threshold with top SNP was 250 kilobase. 

All analyses were run according to additive, dominant and recessive models. Population, age and gender were covariates in all Plink logistic and linear regression models. Additionally, when testing an SNP × CHA/RLE interaction effect, the main effects of both the SNP and CHA/RLE were also entered as covariates in the model. Nominal significance threshold was *p* < 0.05. To correct for multiple comparisons in analyses for each of the above outcome variables, Bonferroni correction was applied. The design and methods of our study are shown in Figure 1.

## 5. Conclusions

In conclusion, we found that variation along the *P2RX7* gene encoding the purinoceptor P2X7 is associated with markers of increased risk of suicide and mediates the effects of early childhood adverse experiences and traumas on later risk of suicide. Our study highlights both our limited understanding of the pathophysiology of suicide, and the need to identify additional brain targets and pathways to effectively minimize the heavy burden of suicide. Exploring neuroinflammatory pathway and in particular the purinoceptors may provide novel insights into the pathophysiology of different neuropsychiatric disorders, and may yield novel and much-needed biomarkers, such as genetic variations, to guide the treatment of these disorders.

## Data Availability

The data presented in this study are available online in FigShare repository at https://doi.org/10.6084/m9.figshare.24877737.v1.

## Supplementary Materials

The following supporting information can be downloaded at: https://www.mdpi.com/article/10.3390/ijms25020865/s1.
